# The relationship of MicroRNA-21 and plaque stability in acute coronary syndrome

**DOI:** 10.1097/MD.0000000000018049

**Published:** 2019-11-22

**Authors:** Wangwei He, Liyuan Zhu, Yu Huang, Yinfen Zhang, Weimin Shen, Lihuan Fang, Jun Li, Zhuo Wang, Qiang Xie

**Affiliations:** a Department of Cardiology, the First Affiliated Hospital of Xiamen University, Xiamen, Fujian Province; b Department of Cardiology, Renmin Hospital of Wuhan University, Cardiovascular Research Institute of Wuhan University, Wuhan, Hubei Province, China.

**Keywords:** acute coronary syndrome, microRNA-21, optical coherence tomography, vulnerable plaques

## Abstract

Acute coronary syndrome (ACS) leads to clinical symptoms such as chest pain, dyspnea, and arrhythmia. The occurrence of ACS is mainly related to the vulnerable plaques in the coronary arteries. MicroRNA-21 (miR-21) is widely expressed in cardiovascular disease and considered as a marker of myocardial infarction, but its role in vulnerable atherosclerotic plaque of ACS is poorly studied. The cases of ACS and control group were selected in 2 years. Our results revealed that miR-21 was highly positively correlated with the maximum lipid core area, the number of diseased vessels, the number of macrophages, the number of vulnerable plaques, and negatively correlated with the thickness of fiber caps. In the high expression group, the number of coronary artery lesions, the number of vulnerable plaques, the core area of lipid pools and the number of macrophages were significantly higher than those in the low expression group and the middle expression group. But the high expression group of the thickness of the fiber cap was significantly lower than that of the low expression group and the medium expression group. These studies show that miR-21 is an important factor leading to vulnerable plaque instability in ACS, and it can be a predictor of acute adverse events in coronary heart disease.

## 1 Introduction

Acute coronary syndrome (ACS) refers to coronary plaque rupture and thrombosis caused by myocardial tissue ischemia and leads to clinical symptoms such as chest pain, dyspnea, and arrhythmia. It mainly includes acute myocardial infarction and unstable angina pectoris.^[1]^ ACS has high morbidity and sudden death rate. It is a disease that seriously endangers human health. The occurrence of ACS is mainly related to the vulnerable plaques in the coronary arteries.^[2]^ The determinant of plaque vulnerability is not the size of the patch, but the structure of the plaque. At present, it is considered that the fibrous cap (<65 μm), the rich lipid in the plaques, and the infiltration of many macrophages in the vicinity of the plaques are the most important characteristics of vulnerable plaques.^[3–5]^

The current methods used to detect plaque vulnerability are intravascular ultrasound (IVUS), vascular mirror and optical coherence tomography (OCT). OCT imaging technique in coronary artery is a new imaging technique with high split rate. It images biological tissue by near infrared ray, optical interference principle and reflected optical echo.^[6]^ It is the highest resolution imaging technique in current clinical medical images. Compared with angioscopy and IVUS, it is the most effective way to identify vulnerable plaques in the coronary artery in vivo.^[7,8]^

MicroRNAs (miRNAs) are a class of non-coding small RNA molecules with about 22 nucleotides. It inhibits mRNA translation or results in mRNA degradation by binding to the 3’-UTR region of the target gene mRNA, which influences gene expression at the post-transcriptional level and plays an important biological function.^[9]^ Recent studies have found that miRNA-21 (miR-21) is widely expressed in cardiovascular disease.^[10]^ Especially in acute myocardial infarction, the role of miR-21-PTEN/AKT-p38-caspase-3 signaling pathway was used to reduce myocardial infarction, reduce myocardial infarction area, and improve cardiac systolic function.^[11,12]^ MiR-21 can be considered as a marker of myocardial infarction, but its role in predicting vulnerable plaques is poorly studied.

## 2 Materials and methods

### 2.1 Materials

The study protocol was approved by the ethics committee of the First Affiliated Hospital of Xiamen University. All patients provided informed consent. ACS cases were collected from Aug in 2015 to Aug in 2017. All patients were included in the general clinical data, including age, sex, smoking history, history of hypertension, diabetes mellitus and hyperlipidemia (Table 1). All patients were examined by coronary angiography. If there is 1 or more coronary artery stenosis degree >50% is included in the study. Exclusion criteria: no diagnostic criteria for coronary heart disease; patients with complete occlusion of blood vessels; patients with acute ST segment elevation myocardial infarction, renal insufficiency, combined with infectious diseases; patients with contraindications of coronary angiography and contraindications to OCT examination.

**Table 1:**
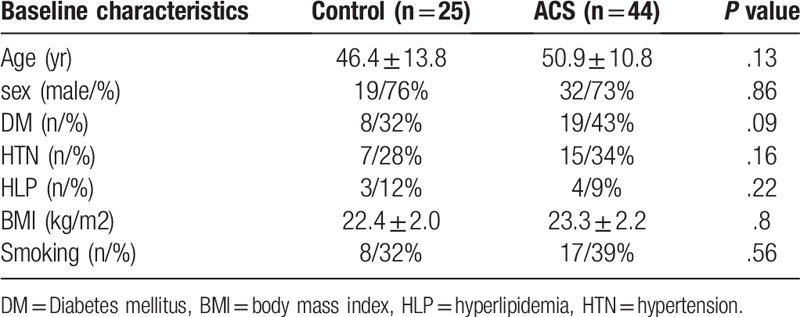
baseline characteristics of both group.

### 2.2 Collection of blood samples

In addition to routine examination, all cases were placed in the EDTA tube at about 5 ml of the elbow vein on the morning of surgery. 4 °C, 3000 r/minute, centrifuge for 15 minutes, and take the supernatant liquid to be stored in the –80 refrigerator. In this study, the expression level of miR-21 was compared with that of healthy volunteers. The expression of miR-21 is 2-ΔCT, ΔCT = ΔCT_miR-21_-ΔCT_β-actino_ΔΔCT = ΔCT_experimental group_-ΔCT_the control group_.

### 2.3 Total RNA extraction and reverse transcription reaction

Trizol LS kit (Invitrogen, USA), RNA reverse transcription kit (Invitrogen, USA), fluorescence real-time quantitative PCR kit (Invitrogen, USA) were used. The miR-21 sequence is: UAGCUUAUCAGACUGAUGUUGA (ABI, USA); β-actin: CACGATGGAGGGGCCGGACTCATC (ABI, USA). The total RNA was extracted from the plasma by the instruction manual. The OD260 and OD280 readings of each sample were measured respectively. OD260/OD280 indicated that the RNA purity was better, and the RNA concentration was detected in 1.8–2.0. The reverse transcriptional reaction of miR-21 and its internal reference was performed by using specific primers (designed and synthesized by Wuhan Bayfield Biological Co, LTD.). Take 500 ng samples for reverse transcription. Reaction conditions: 37 °C for 15 minutes, 85 °C for 5 seconds. PCR amplification was performed with cDNA as template, and the relative expression quantity of each specimen was calculated according to formula 2^-ΔΔCT^. Reaction conditions are as follows: 95 °C for 20 seconds, 95 °C for 10 seconds, 60 °C for 15 seconds, 70 °C for 10 seconds, cycle of 50 times.

### 2.4 Research groupings

The outpatients or inpatients who underwent coronary angiography with coronary artery stenosis degree <30% were defined as controls, a total of 25 cases. In the selected ACS patients, according to the miR-21 expression level, the patients with miR-21 < 7 (6.173 + 0.160, n = 15) were defined as low expression group. 7 < miR-21 < 10 (8.326 + 0.195, n = 21) was defined as the middle expression group. miR-21 > 10 (10.80 + 0.278, n = 8) was defined as a high expression group.

### 2.5 OCT check

Coronary angiography was performed by means of a radial or femoral artery. A guide catheter with type 6F was used to reach the coronary artery. The OCT imaging catheter is placed in the distal part of the coronary artery. Direct injection of contrast agent (1–3 ml/second) was used to clear the blood flow in the coronary artery, and automatic retracting device at 1 mm/second was used to complete the imaging of the lesion.^[13]^ If there are multiple vessel diseases at the same time, OCT examination is needed. The number of vulnerable plaques was measured, the thickness of the fiber cap of each vulnerable plaque was measured, and the percentage of the largest area of the lipid core in the area of the plaque was measured. On the computer, the images of vulnerable plaques were enlarged, and the macrophages were counted. If there are several cases, the fiber cap is discontinuous, the plaque is formed in the plaque, although there is no whole fibrous cap rupture but the surface membrane breakage or endothelial cell layer is missing, which is defined as the fibrous cap rupture, and the thickness of the fibrous cap is zero. The thickness of the fiber cap is defined as the minimum distance from the coronary artery to the boundary of the lipid pool. The number of macrophages was counted in the 2 quadrants of the same vulnerable plaque. The average number of macrophages was the number of macrophages in a vulnerable plaque. The sum of macrophages in all vulnerable plaques is the total number of macrophages. The above quantitative indicators were measured 3 times, and the average value was taken as the final measurement result.

### 2.6 Statistical analysis

SPSS22.0 statistical software package was used for statistical analysis. Count data were compared by *χ*^2^ test. The measurement data were expressed by mean ± standard deviation (χ ± s). The *t* test was used between the 2 groups, and the difference between *P* < .05 was statistically significant. The PLS (Partial least squares) model was established by using SIMCA-p12.0 software. The number of damaged vessels, the number of vulnerable plaques, the thickness of the fiber cap, the maximum area of lipid core (%), the number of macrophages were set as the dependent variable *y*, and the other indexes (hs-CRP, miR-21, sex, age, diabetes and so on) were set as independent variables. The model R2Y (cum) 0.773 and Q2Y (cum) are 0.726, indicating that the model has a good degree of interpretation and the degree of distinction (R2 and Q2 refer to the model interpretation rate and prediction accuracy, when the cumulative amount of both is close to 1, it means that the model is ideal, with good release rate and prediction rate).

## 3 Results

### 3.1 The baseline features of the patient

**Figure 1:**
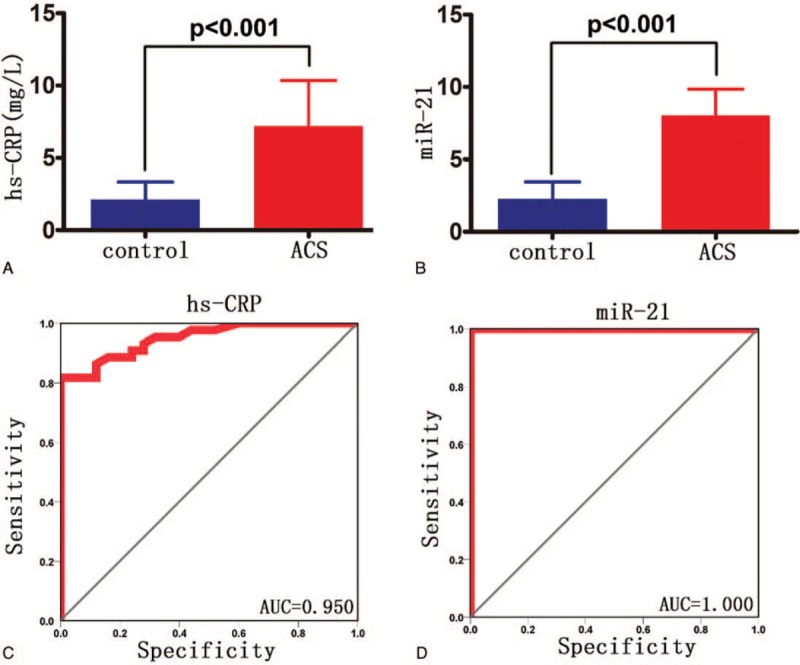
The levels of miR-21 and hs-CRP in ACS group were significantly higher than those in healthy controls (*P* < .001, A and B). A. hs-CRP expression levels in both groups; B. expression levels of miR-21 in both groups; The levels of miR-21 and hs-CRP could distinguish healthy controls and ACS patients and the area under ROC curve (AUC) reached 0.950 and 1, respectively. C. hs-CRP ROC curve; D. miR-21 ROC curve. Control: healthy control group; ACS: ACS group.

The levels of miR-21 and hs-CRP in ACS group were significantly higher than those in healthy controls (*P* < .001, Fig. 1Aand B). Through the receiver operating characteristic curve (ROC curve) analysis, miR-21 and hs-CRP could distinguish healthy controls and ACS patients (Fig. 1C and D), and the area under ROC curve (AUC) reached 0.950 and 1, respectively and had higher diagnostic value.

### 3.2 Correlation analysis of vulnerable plaque

**Figure 2:**
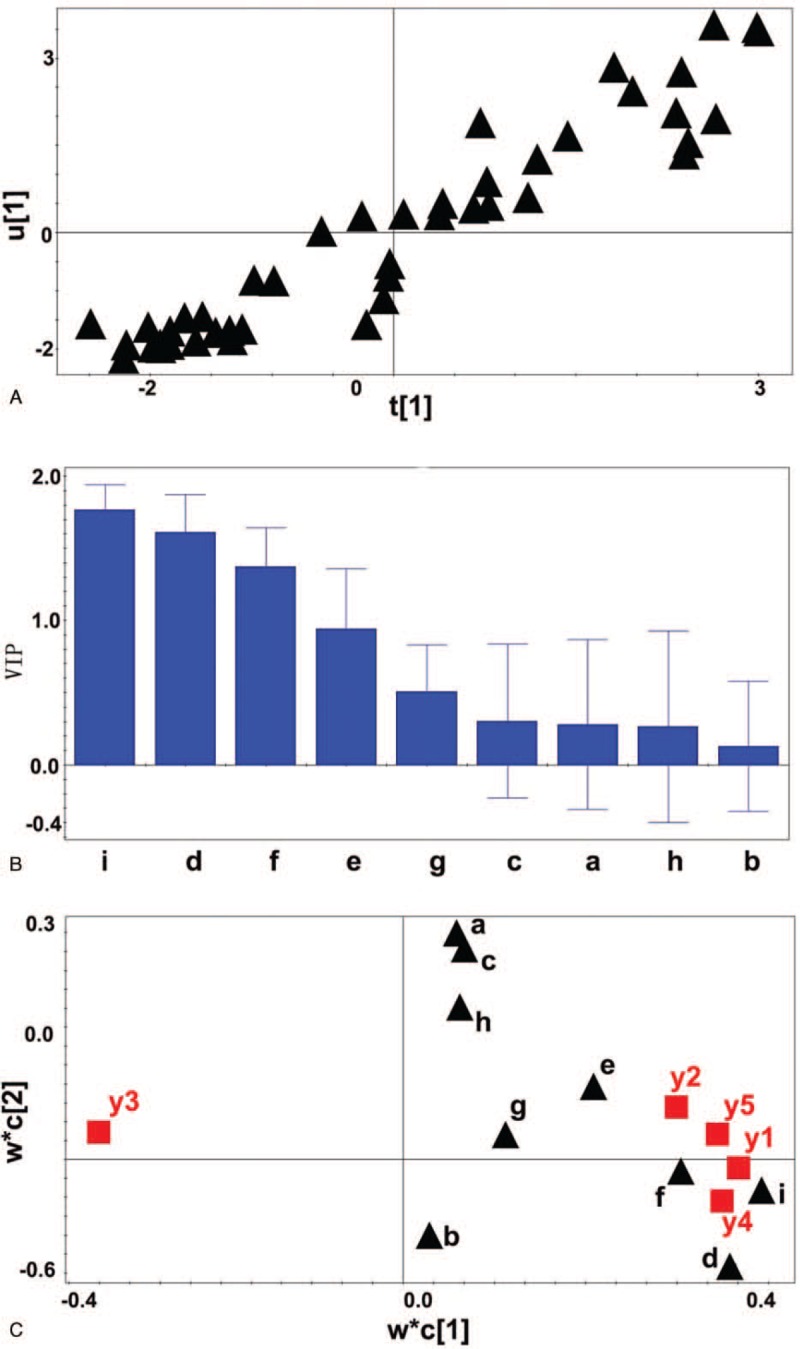
A linear relationship between the number of vascular lesions, the number of vulnerable plaque, the thickness of the fibrous cap, the maximum area of the lipid core (%), the number of macrophages (Y variable) and the other explanatory variables (indexes as independent variables). A: t[1]∗u[1] scatter plot. B: VIP value of relevant observation indicators. C: w∗c1/w∗c2 scatter diagram. a: gender b: age c: weight d: hs-CRP e: hypertension f: diabetes g: hyperlipidemia h: smoking I: miR-21 expression level. Y1: the number of coronary lesions y2: vulnerable plaque number y3: fiber cap thickness y4: maximum lipid core area y5: the number of macrophages.

The t1/u1 map shows that there is a clear linear relationship between the number of vascular lesions, the number of vulnerable plaque, the thickness of the fibrous cap, the maximum area of the lipid core (%), the number of macrophages and the other explanatory variables (Fig. 2A), which indicates that the establishment of the model is reasonable. In the PLS model, the variable importance in the projection (VIP) value greater than 1 is the key variable and contributes greatly to the *y* value. In this study, hs-CRP, miR-21, and diabetes (VIP > 1) were highly correlated with the maximum area of lipid core, the number of vessels, the number of macrophages, the thickness of the fibrous cap and the number of vulnerable plaques. miR-21 correlation is the highest among them (Fig. 2B). The w∗c[1]/ w∗c[2] map shows that hs-CRP, miR-21 and diabetes are highly correlated with the maximum area of lipid core, the number of diseased vessels, the number of macrophages, and the number of vulnerable plaques (Fig. 2C). These indexes are highly negatively correlated with the thickness of fiber caps.

### 3.3 The relationship between miR-21 and the index of vulnerable plaque

**Figure 3:**
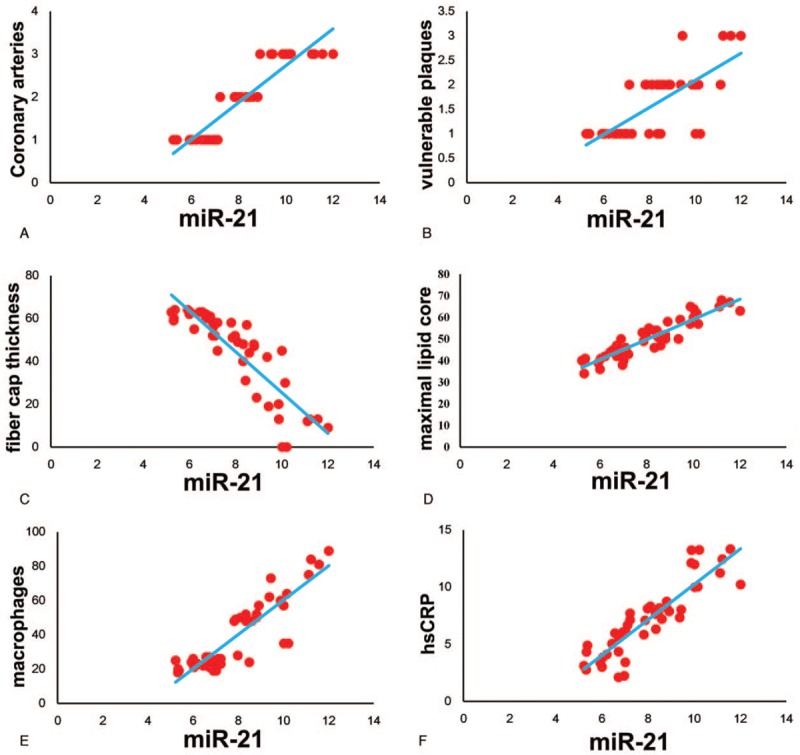
The expression level of miR-21 is positively correlated with the number of coronary artery lesions (A), the number of vulnerable plaques (B), the area of the lipid core area (D), the number of macrophage infiltration (E) and the level of hs-CRP expression in plasma (F). The expression level of miR-21 was negatively related to the thickness of vulnerable cap's fibrous cap (C).

By scatter plot analysis, we can see that the expression level of miR-21 is positively correlated with the number of coronary artery lesions (Fig. 3A). At the same time, we can see that the expression level of miR-21 is positively correlated with the number of vulnerable plaques (Fig. 3B), the area of the lipid core area (Fig. 3D), the number of macrophage infiltration (Fig. 3E) and the level of hs-CRP expression in plasma (Fig. 3F). At the same time, miR-21 was negatively related to the thickness of vulnerable cap's fibrous cap (Fig. 3C).

### 3.4 The relationship between miR-21 and coronary angiography and OCT examination results

**Figure 4:**
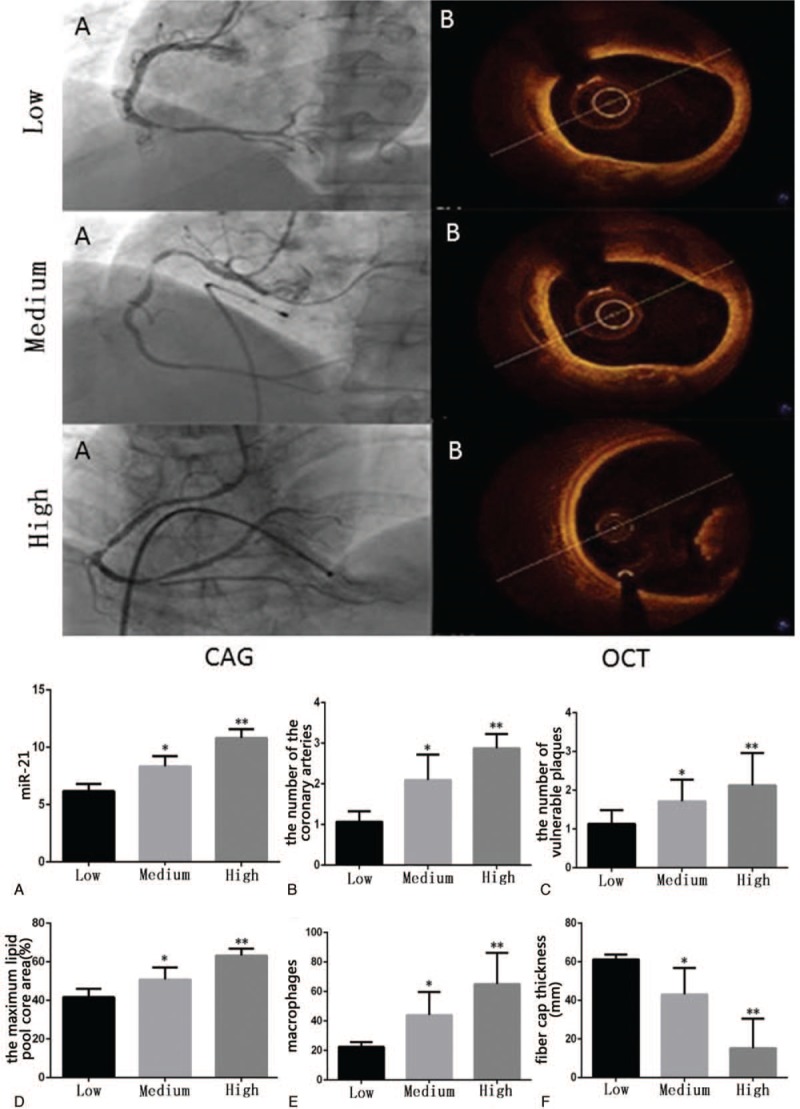
MiR 21 expression level with coronary angiograph (Upper part A) and OCT examination (Upper part B). Bottom part: A: the expression of miR-21 was divided into groups. The number of coronary artery lesions (B), the number of vulnerable plaque (C), the core area of the lipid pool (D), and the number of macrophages (E) were significantly higher than those in low expression group. The thickness of fiber cap was significantly lower than that of low expression group (F). CAG: imaging of coronary angiography; OCT: OCT imaging. Low: Low expression group (miR-21 < 7); Medium: Medium expression group (7 < miR-21 < 10); High: High expression group (miR-21 > 10). CAG: coronary angiography; OCT: optical coherence tomography; Low: Low expression group; Medium: Medium expression group; High: High expression group. ∗ *P* < .01 vs Low. ∗∗ *P* < .01 vs Low.

The effects of three groups of different miR-21 levels on the number of coronary artery lesions, the number of vulnerable plaques, the core area of lipid pool, the number of macrophages, and the thickness of the fibrous cap were analyzed by the results of coronary angiography and OCT examination (Fig. 4A(CAG), 4B(OCT)). In the high expression group, the number of coronary artery lesions (2.875 ± 0.125 vs 1.067 ± 0.067, *P* < .01), the number of vulnerable plaque (2.125 ± 0.295 vs 1.133 ± 0.091, *P* < .01), the core area of the lipid pool (63.25 ± 1.278 vs 41.67 ± 1.124, *P* < .01), and the number of macrophages (65.00 ± 7.510 vs 22.47 ± 0.822, *P* < .01) All of them were significantly higher than those in low expression group (Fig. 4A–E). The thickness of the fiber cap in the high expression group was significantly lower than that in the low expression group (15.25 ± 5.391 vs 61.27 ± 0.621, *P* < .01) and the medium expression group (15.25 ± 5.391 vs 43.10 ± 3.003, *P* < .01) (Fig. 4F). Compared with the low expression group, the number of coronary arteries (2.095 ± 0.136 vs 1.067 ± 0.067, *P* < .01), the number of vulnerable plaque (1.714 ± 0.1223 vs 1.133 ± 0.091, *P* < .01), the core area of the lipid pool (50.81 ± 1.353 vs 41.67 ± 1.124, *P* < .01), and the number of macrophages (44.05 ± 3.421 vs 22.47 ± 0.822, *P* < .01) increased significantly (Fig. 4A–E). The thickness of fiber cap was significantly lower than that of low expression group (43.10 ± 3.003 vs 61.27 ± 0.621, *P* < .01) (Fig. 4F).

## 4 Discussion

The essence of coronary atherosclerosis is the chronic inflammatory reaction of continuous smooth muscle cells and collagen fibrils based on long-term lipid or blood composition deposition.^[14]^ Vulnerable plaque is the main cause of ACS. Inflammatory activation and thin cap fibroatheroma (TCFA) with large lipid core, thrombus formation and plaque rupture are the main features of vulnerable plaque.^[15]^ The exact mechanism of how to activate the inflammatory response when ACS occurs and how the inflammatory response promotes plaque rupture is still controversial.

In recent years, microRNAs have been found to play an important role in the regulation of inflammation. It is widely used in eukaryotes and is widely involved in the regulation of cell proliferation, differentiation, tumor formation, immune function, and other physiological and pathological processes. The role of miR-21 in cardiovascular system has been widely concerned. Studies have shown that it is related to the degree of coronary artery stenosis, but all these studies are all studied by coronary angiography or coronary artery CTA. For the first time, we studied the relationship between miR-21 and unstable plaques by OCT. In this study, we found that the expression level of miR-21 has a significant correlation with several common indicators of plaque instability, such as the thickness of the fibrous cap, the size of the largest core area of the lipid pool, and the number of macrophages. The higher the expression level of miR-21, the thinner the thickness of the fibrous cap, the larger the core area of the largest lipid pool and the more the number of macrophages around the fiber cap, suggesting that the plaque is more unstable. It is proved that miR-21 can be used as a detection method for plaque stability.

Our study confirmed that there is a significant linear relationship between hs-CRP and vulnerable plaques, whether in quantity, thickness of fibrous cap, or the number of macrophages, and the core area of the lipid pool. It is proved again that inflammation is one of the key factors that lead to plaque vulnerability. Under the action of the oxidative stress reaction inside and outside the vessel and the infection factor, the liver is induced to synthesize hs-CRP, triggering the inflammatory cascade reaction, making many mononuclear cells infiltrating into the plaque and transforming into macrophages. MiR-21 itself has the role of regulating the inflammatory network.^[16]^ The infiltration of macrophages to the cap can destroy the integrity of the fibrous cap by secreting matrix metalloprotease-1 (MMP-1), matrix metalloprotease-9 (MMP-9) and so on, which can cause the fibrous cap to be thinner, resulting in plaque rupture and thrombosis.^[17]^ OCT measurement of macrophage density is highly correlated with histological findings. It is the only way to quantify macrophages in fiber caps in vivo.^[2]^ We used the high-resolution test of OCT to confirm that there are a large number of macrophages around vulnerable plaques in ACS patients, and the more the number indicates the more unstable the plaque is.

Besides, miR-21 has many target genes, but mainly in SPRY1 (sprout homologue 1), PTEN (phosphatase and tensin homologue) and other genes in cardiovascular diseases.^[18]^ In acute myocardial ischemia, miR-21 can inhibit PTEN gene expression and promote matrix MMP-2 expression. MMP-2 has the function of degrading extracellular matrix, destroying the integrity of fibrous cap, resulting in plaque rupture.^[19]^ It can be seen that at ACS, miR-21 can not only indirectly induce inflammatory response, but also affect the coronary plaque by direct targeting gene regulation. Recent study reported that measurement of circulating levels of miR-21 could differentiate patients with Heart Failure of ischemic etiology from those with a non-ischemic etiology.^[20]^ We suspect that it could be useful as a biomarker for the triage of patients presenting with an ACS.

## 5 Conclusion

In conclusion, the expression level of miR-21 was highly consistent with the levels of hs-crp expression levels in plasma and the number of macrophages infiltrating in vulnerable plaques. We believe that miR-21 is an important factor leading to plaque instability. The higher the expression level of miR-21, the more unstable the plaque, the higher the risk of cardiovascular events. This means that miR-21 can be a predictor of acute adverse events in coronary heart disease in the future. We suspect that the leading cause of plaque instability mechanism on the one hand, inflammation cells infiltration, the release of inflammatory medium leads to dissolve the fibrous cap, leading to thinning the thickness of fibrous cap, plaque rupture and thrombosis related to outside, on the other hand may also part instead of directly targeting regulatory genes. We will observe the effect of miR-21 gene deletion on inflammatory factors and plaque stability in the next experiment to confirm its role in vulnerable plaque. We believe that miR-21 might be a useful therapeutic target in the setting of vulnerable patients and a novel drug to inhibit miR-21 should be developed in the future.

## Acknowledgments

Kaimin Lin, Lei Gao, and Junyu Han are gratefully acknowledged for providing technology help.

## Author contributions

**Conceptualization:** Wangwei He, zhuo Wang.

**Data curation:** Liyuan Zhu, Yinfen Zhang, Lihuan Fang, Jun Li.

**Formal analysis:** Yinfen Zhang, Jun Li.

**Funding acquisition:** Wangwei He, Qiang Xie.

**Investigation:** Wangwei He, Weimin Shen, zhuo Wang.

**Methodology:** Liyuan Zhu, Weimin Shen, Lihuan Fang.

**Project administration:** Liyuan Zhu, Yu Huang, zhuo Wang.

**Resources:** Liyuan Zhu, Yu Huang, Weimin Shen.

**Software:** Yu Huang, Weimin Shen.

**Supervision:** Qiang Xie.

**Writing – review & editing:** Wangwei He, Qiang Xie.

**Writing – original draft:** zhuo Wang, Qiang Xie.
